# Inhibition of Rheumatoid Arthritis Using Bark, Leaf, and Male Flower Extracts of *Eucommia ulmoides*

**DOI:** 10.1155/2020/3260278

**Published:** 2020-08-13

**Authors:** Yun-Yun Xing, Jian-Ying Wang, Kai Wang, Yan Zhang, Kun Liu, Xiao-Yun Chen, Ying Yuan

**Affiliations:** ^1^School of Pharmacy, Shanghai University of Traditional Chinese Medicine, No. 1200 Cailun Road, Pudong District, Shanghai, China; ^2^Shanghai Innovation Center of TCM Health Service, Shanghai University of Traditional Chinese Medicine, No. 1200 Cailun Road, Pudong District, Shanghai, China; ^3^Science and Technology Experiment Center, Shanghai University of Traditional Chinese Medicine, No. 1200 Cailun Road, Pudong District, Shanghai, China; ^4^Longhua Hospital, Shanghai University of Traditional Chinese Medicine, Shanghai, China

## Abstract

*Eucommia ulmoides* Oliv., a native Chinese plant species, has been used as a traditional Chinese medicine formulation to treat rheumatoid arthritis (RA), strengthen bones and muscles, and lower blood pressure. Various parts of this plant such as the bark, leaves, and flowers have been found to have anti-inflammatory properties. *E. ulmoides* has potential applications as a therapeutic agent against bone disorders, which were investigated in this study. *In vitro*, RA joint fibroblast-like synoviocytes (RA-FLS) were treated with different concentrations (0, 25, 50, 100, 200, 400, 800, and 1000 *μ*g/mL) of *E*. *ulmoides* bark, leaf, and male flower alcoholic extracts (EB, EL, and EF, respectively) to determine their potential cytotoxicity. Tumor necrosis factor- (TNF-) *α* and nitric oxide (NO) levels in RA-FLS were quantified using enzyme-linked immunosorbent assay (ELISA). Furthermore, collagen-induced arthritis (CIA) rats were treated with EB, EL, EF, *Tripterygium wilfordii* polyglycoside (TG) or the normal control (Nor), and then ankle joint pathology, bone morphology, and serum and spleen inflammatory cytokine levels were evaluated. The results showed that, in RA-FLS, EB, EL, and EF were not cytotoxic; EB and EF reduced TNF-*α* supernatant levels; and EB, EL, and EF reduced NO levels. The results of *in vivo* experiments showed that EB, EL, and EF alleviated ankle swelling and joint inflammation, while all extracts diminished inflammatory cell infiltration, pannus and bone destruction, and bone erosion. All tested extracts inhibited interleukin- (IL-) 6, IL-17, and TNF-*α* mRNA in the spleen of CIA rats, while EB most effectively reduced osteoclasts and inhibited bone erosion. EF showed the most obvious inhibition of inflammatory factors and pannus. Thus, EB, EL, and EF may alleviate bone destruction by inhibiting inflammation.

## 1. Introduction

Rheumatoid arthritis (RA) is a chronic autoimmune disease mainly characterized by synovial inflammation and bone destruction [1]. Fibroblast-like synoviocytes (FLS) in normal synovial tissues maintain normal joint function by producing extracellular matrix components and joint lubricants. However, in RA, FLS can be induced by immunopathological mechanisms and signal transduction to synthesize numerous factors that mediate inflammation and joint damage [2]. Inflammatory cytokines including tumor necrosis factor- (TNF-) *α*, interleukin- (IL-) 1*β*, IL-6, and IL-17 play a critical role in the pathogenesis of RA. Specifically, TNF-*α*, IL-1*β*, and IL-17 induce RA synovitis with the promotion of vasospasm and cause bone destruction in late-stage synovitis [3].

Four main approaches are used for the treatment of RA: nonsteroidal anti-inflammatory drugs, disease-modifying antirheumatic drugs, glucocorticoids, and herbal preparations [4]. However, none of the new therapeutic drugs show long-term alleviation of the symptoms and effects in most RA patients. Additionally, many treatments cause adverse reactions. Therefore, identifying effective and low-toxicity therapeutic drugs from botanical sources is a current research interest and focus.

The barks of *Eucommia* species have a long history of medicinal use and modern research has identified the presence of lignans, iridoids, phenylpropanoids, and flavonoids, as well as several amino acids and trace elements in the barks of these species [5–7]. *Eucommia* bark has blood pressure and fat lowering, hypoglycemic, antifatigue, antiosteoporosis [8–12], and other pharmacological effects [13, 14], but it is often in short supply owing to its slow growth. *Eucommia ulmoides* leaves and bark have similar active ingredients (chlorogenic acid and aucubin) and pharmacological effects (such as anti-inflammatory and blood pressure lowering) [15]. In addition, the male flowers of this species contain abundant proteins, amino acids, and total flavonoids [16]. The male flowers of *E. ulmoides* have higher nutritional value and health-promoting effects than the bark and leaves.

The male flowers of *E. ulmoides* are relatively abundant and cost less than the bark; therefore, their development and utilization prospects are better. Our research group previously found that *Eucommia* bark, leaves, and male flowers have relatively similar anti-inflammatory, analgesic, bacteriostatic, and immunomodulatory activities and other pharmacodynamic properties [13]. The alcoholic extract of *E. ulmoides* inhibited the proliferation of synovial cells in a collagen-induced arthritis (CIA) rat model and production of serum and tissue inflammatory cytokines such as TNF-*α* and IL-1*β* while reducing cartilage and bone destruction [14].

To comparatively explore the effects of *Eucommia* bark, leaf, and male flower alcoholic extracts (EB, EL, and EF, respectively) on RA bone destruction, we used type collagen in this study, to establish a CIA rat arthritis model, which was confirmed to have been successfully established based on the degree of foot plantar swelling, histopathology, imaging, and analysis of proinflammatory factors. The inhibitory effects and potency of EB, EL, and EF on joint inflammation and bone destruction were comparatively evaluated.

## 2. Materials and Methods

### 2.1. Ethanol Extracts of the Plant Material

*E. ulmoides* bark, leaves, and male flowers were purchased from Shanghai Kangqiao Chinese Medicine Tablet Co., Ltd. (Shanghai, China) and were authenticated by Professor Wu Jinrong of the Shanghai University of Traditional Chinese Medicine. Plant materials were extracted twice using 70% ethanol (1 : 8, w/v for 1 h; Sinopharm Chemical Reagent Co., Ltd., Shanghai, China) and filtered. The EB, EL, and EF were then concentrated (1 g/mL) *in vacuo* [17, 18].

### 2.2. Cell Culture and 3-(4,5-Dimethylthiazol-2-yl)-2,5-diphenyltetrazolium Bromide (MTT) Assay

The human MH7A RA fibroblast synovial cell line (Biovector NTCC, Beijing, China) was cultured in Dulbecco's modified Eagle's medium (DMEM) supplemented with 10% fetal bovine serum (FBS) and 1% antibiotics (Gibco, CA, USA) in an atmosphere of 5% CO_2_ at 37°C. Exponential (logarithmic) phase RA-FLS were seeded in a 96-well plate at a density of 2,000 cells/well in 200 *μ*L medium and incubated overnight. Then, the medium was aspirated and 10% FBS and varying concentrations (0, 25, 50, 100, 200, 400, 800, and 1000 pg/mL) of EB, EL, and EF were added. Each extract-treated group had six replicate wells and the plates were placed in a CO_2_ incubator at 37°C for 48 h. Then, the medium was removed and 140 *μ*L MTT working solution (5 mg/mL) was added, followed by incubation for 4 h. The supernatant was discarded and dimethyl sulfoxide (DMSO) was added, followed by determination of the optical density (OD) at 490 nm using a spectrophotometer. The cell survival rate was calculated using the following formula: cell survival rate (%) = (experimental group OD-zero adjustment group OD)/(control group OD-zero adjustment group OD) × 100.

### 2.3. Determination of TNF-*α* and NO in RA-FLS Supernatant

RA-FLS were seeded in 96-well plates at a density of 2,000 cells in aliquots of 100 *μ*L/well and treated with 500 *μ*g/mL EB, EL, EF, or *Tripterygium wilfordii* polyglycoside (TG, control). Blank samples contained a standard growth medium (negative control). After 48 h of treatment, the RA-FLS extracellular culture medium was collected for each group and TNF-*α* and NO levels were measured using enzyme-linked immunosorbent assay (ELISA) kits (Hangzhou, China) as described previously [19].

### 2.4. CIA Model and Extract Administration

An emulsion of type II collagen (CII) and incomplete Freund's adjuvant (CFA, Sigma Aldrich, St. Louis, MO, USA) was prepared as follows: on day 1, acetic acid solution (0.1 mol/L) was prepared, precooled at 4°C, mixed with bovine CII (Sigma Aldrich), and stored overnight at 4°C. On day 2, the mixture was emulsified with an equal volume of CFA on ice to obtain a stable CII/CFA emulsion [14]. Wistar rats (female : male = 1 : 1) weighing 100 ± 10 g were purchased from Beijing CRL Laboratory Animal Co., Ltd. (China) and adaptively fed for 3 days in the Laboratory Animal Center of Shanghai University of Traditional Chinese Medicine.

On day 0 and day 7, subcutaneous injections of CII/IFA emulsion were administered to 40 rats through the tail and into the back (0.1 mL each). Eight randomly selected rats were not administered the arthritis-inducing treatment and constituted the normal group (Nor; four male and four female rats), which was mock-treated with distilled water. The CIA (negative control) group was treated with distilled water while the EB, EL, and EF groups were each given the respective extract at 4 g·kg^−1^ day^−1^ [20]; the positive control TG group received a dose of 6 mg·kg^−1^ day^−1^ [21]. The treatments were administered intragastrically (i.g.) according to the equivalent dose conversion between animals once daily from day 14 for 4 weeks.

### 2.5. Rat Foot Swelling and Arthritis Evaluation

After the first collagen injection, hind-paw swelling and volume were measured once weekly using a paw volume meter (YLS-7B; Jinan Yiyan Science and Technology Development Co., Ltd. Shandong, China). The clinical severity of RA in rat paws was scored using a subjective scale with a range of 4–0: 4, joint deformity; 3, pronounced swelling; 2, moderate swelling; 1, mild swelling; and 0, normal. The final joint score was the average value of the two hind limbs of each rat. Arthritis scoring was performed by three independent observers who were blinded to the assay design [22].

### 2.6. Sample Collection

On day 42, the animals were euthanized, blood was collected from the carotid artery, and the spleens were harvested for further analysis. The ankle joint and hind paw were removed and perfused with ice-cold phosphate-buffered saline (PBS). The left hind paws were fixed in 10% formalin for 2 days, whereas the paraformaldehyde-fixed right hind paws were separately placed in 70% ethanol for 1 day.

### 2.7. Histopathological Analysis

Histopathological analysis was conducted to determine the effects on arthritis and synovitis, pannus formation, degradation of cartilage, and for bone analysis. Briefly, rat ankle joints were decalcified in 0.5 M ethylenediaminetetraacetic acid (EDTA, pH 7.2) for 4 weeks and paraffin-embedded, and then the tissue was cut into 5 *μ*m sections using a fully automated paraffin slicer (Leica Microsystems, Wetzlar, Germany). The formalin-fixed sections were stained using hematoxylin and eosin (H&E) and pathological changes were assessed using microscopy.

H&E-stained slides were scored by three independent observers who were blinded to the assay design. Cellular infiltration was scored on a scale from 0 to 3: 0, none; 1, mild; 2, moderate; and 3, severe infiltration. Pannus formation was also scored from 0 to 3: 0, none; 1, mild; 2, moderate; and 3, severe pannus. The degradation score of cartilage and bone used a scale ranging from 0 to 3: 0, no moderate degradation or joint-space narrowing; 1, mild degradation without joint-space narrowing; 2, moderate degradation causing moderate joint-space narrowing; and 3, severe degradation causing joint-space narrowing or merging [22].

### 2.8. Microcomputed Tomography (CT) Scanning

Bone erosion in the distal thigh ankle joint was determined using high‐resolution micro‐CT (Skyscan 1176; Bruker, Billerica, MA, USA) scanning at 50 kV tube voltage and 500 *μ*A current. Then, the three‐dimensional (3D) structure of the hind paws was reconstructed using Mimics 18.0 software (Materialise, Leuven, Belgium).

### 2.9. ELISA

Serum IL-6, IL-1*β*, TNF-*α*, and NO levels, along with synovial extracellular fluid TNF-*α* and NO levels, were measured using an ELISA kit according to the manufacturer's protocol.

### 2.10. Real-Time Quantitative Reverse Transcription-Polymerase Chain Reaction (qRT-PCR)

Total RNA from spleen tissue was extracted with TRIzol reagent (Thermo Fisher Scientific, Waltham, MA, USA) using a high-throughput tissue grinder (Shanghai Wonbio Technology Co., Ltd., Shanghai, China). cDNA was synthesized using the PrimeScript reverse transcription (RT) Master Mix (Takara Bio, Shiga, Japan) according to the manufacturer's protocol and used as a template for quantitative RT polymerase chain reaction qRT-PCR using TB Green Premix Ex Taq (2×, Takara Bio, Shiga, Japan). Data were analyzed using the 2^ΔΔCT^ method and normalized to glyceraldehyde 3-phosphate dehydrogenase (GAPDH) as an endogenous reference. The following primers were used IL-6, 5′-CACAGAGGATACCACCCACA-3′ and 5′-CAGAATTGCCATTGCACAAC-3′; IL-17, 5′-GCCGAGGCCAATAACTTTCT-3′ and 5′-GAGTCCAGGGTGAAGTGGAA-3′; TNF-*α*, 5′-GGAAAGCATGATCCGAGATG-3′ and 5′-CGAGCAGGAATGAGAAGAGG-3′; and GAPDH, 5′-CCACCCATGGCAAATTCCATGGCA-3′ and 5′-TCTAGACGGCAGGTCAGGTCCACC-3′.

### 2.11. Statistical Analysis

Intergroup comparisons were performed using a one‐way analysis of variance (ANOVA) followed by Dunnett's *t*-test. Statistical analyses were performed using the statistical package for the social sciences (SPSS) 22.0 (SPSS Inc., Chicago, IL, USA). Data are expressed as means ± standard deviation and a *P* < 0.05 was considered statistically significant.

### 2.12. Ethical Approval

All animal procedures were performed according to the ethical guidelines of the Laboratory Animal Welfare and Animal Experimental Ethics Committee of Shanghai University of Traditional Chinese Medicine (Approval number: SZY201704008).

## 3. Results

### 3.1. RA-FLS Proliferation and Determination of TNF-*α* and NO in Culture Medium

**Figure 1:**
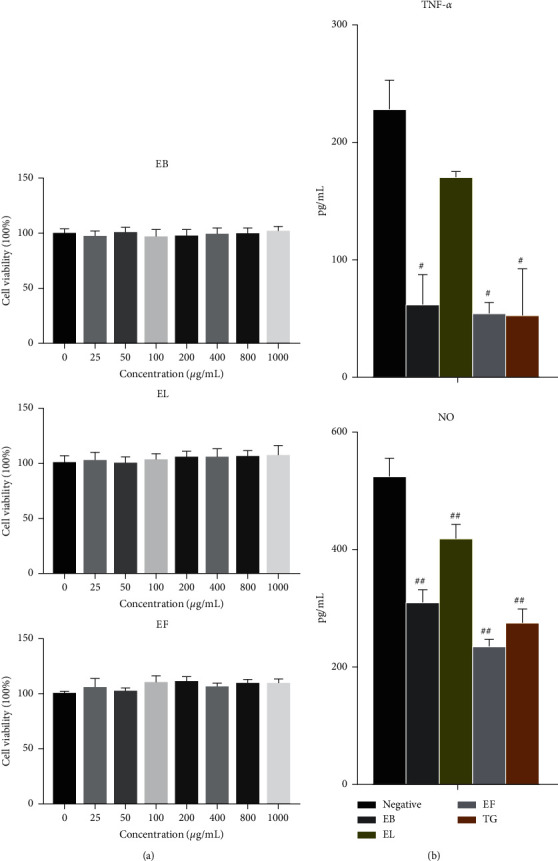
Effects of different *Eucommia ulmoides* extracts on rheumatoid arthritis joint fibroblast-like synoviocytes (RA-FLS, *n* = 6). (a) RA-FLS proliferation in each group. (b) Tumor necrosis factor- (TNF-) *α* and nitric oxide (NO) levels in cell culture supernatants of each group (*n* = 6). ^#^*P* < 0.05 and ^##^*P* < 0.01 compared to the negative control group.

Different concentrations of EB, EL, and EF exhibited no inhibitory effects on RA-FLS cells, indicating that concentrations up to 1000 *μ*g/mL were not cytotoxic (Figure 1(a)). Therefore, experimental groups treated with 500 *μ*g/mL were analyzed for effects on TNF-*α* and NO levels of synovial extracellular fluid. Results showed that TNF-*α* expression of the cell-culture supernatants of the EB, EF, and TG groups decreased significantly (*P* < 0.05) compared to that of the negative control group, while NO expression levels in the cell culture supernatants of the EB, EL, EF, and TG groups decreased significantly (*P* < 0.01) compared to those of the negative control group (Figure 1(b)).

### 3.2. Effects of *E. ulmoides* Extracts on RA Progression

**Figure 2:**
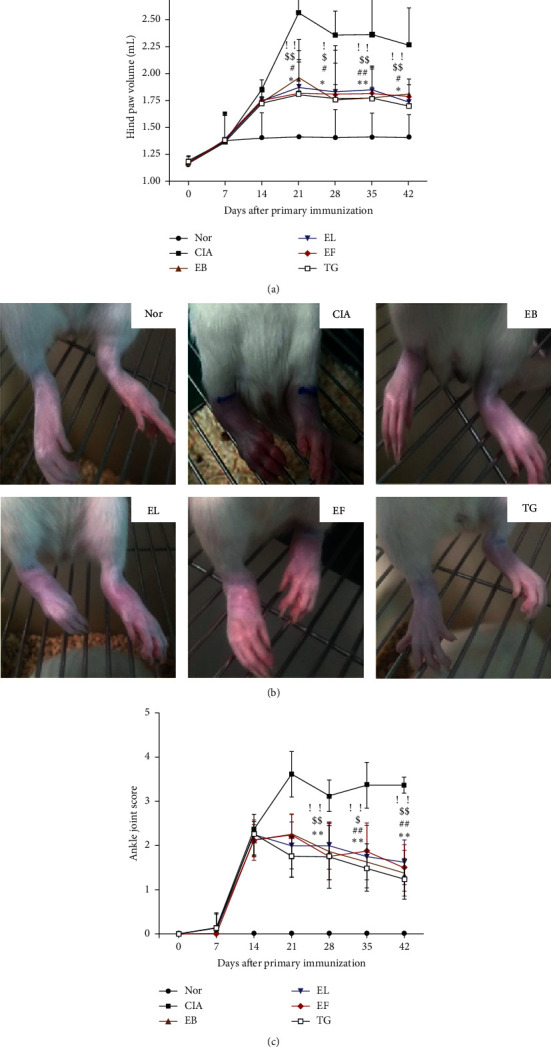
Antiarthritic effect of Eucommia ulmoides extracts in type II collagen-induced arthritis (CIA) rats. (a) Hind paw swelling in CIA rats at different time points, ^*∗*^*P* < 0.05 and ^*∗∗*^*P* < 0.01 (EB) compared with CIA; ^#^*P* < 0.05 and ^##^*P* < 0.01 (EL) compared with CIA; ^$^*P* < 0.05 and ^$$^*P* < 0.01 (EF) compared with CIA; and ^!^*P* < 0.05 and ^!!^*P* < 0.01 (TG) compared with CIA (*n* = 8). (b) Foot and plantar swelling of rats in each group after last administration. (c) Arthritis score of rats in each group, ^*∗*^*P* < 0.05 and ^*∗∗*^*P* < 0.01 (EB) compared with CIA; ^#^*P* < 0.05 and ^##^*P* < 0.01 (EL) compared with CIA; ^$^*P* < 0.05 and ^$$^*P* < 0.01 (EF) compared with CIA; and ^!^*P* < 0.05 and ^!!^*P* < 0.01 (TG) compared with CIA (*n* = 8).

On day 14, foot volume and arthritic score of the rat model showed no significant difference compared to those of the Nor group (*P* > 0.05, Figures 2(a) and 2(c)). On days 21, 28, 35, and 42, the foot volume of the treatment groups was significantly lower than that of the CIA group (*P* < 0.05 or *P* < 0.01, Figure 2(a)). Images of the hind paws of rats from all groups clearly demonstrate the edema-mitigating effects of EB, EL, EF, and TG treatments (Figure 2(b)). On days 28, 35, and 42, arthritic scores of the treatment groups were significantly lower than those of the CIA group (*P* < 0.05 or *P* < 0.01, Figure 2(c)).

### 3.3. Histopathological Examination of the Ankle Joint

**Figure 3:**
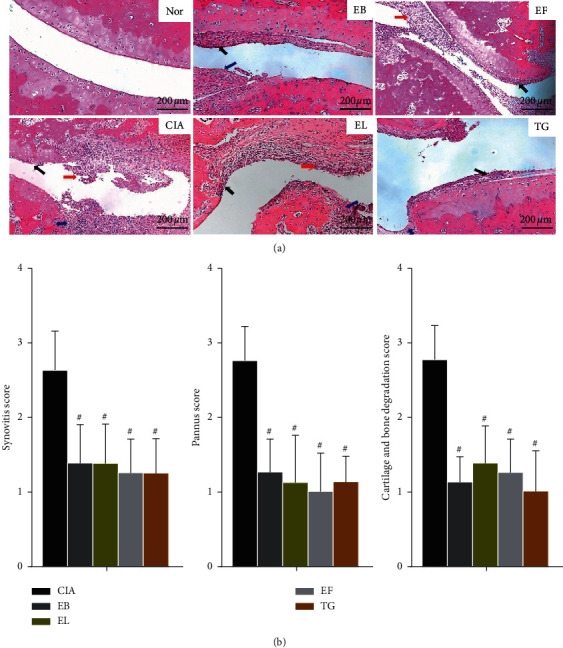
Photomicrographs of pathological lesions of ankle joints of treated and untreated rats. (a) Hematoxylin and eosin (H&E) staining of the ankle joints of collagen-induced arthritis (CIA) rats treated with various extracts (×200). Black arrow, pannus; red arrow, inflammatory cell infiltration; blue arrow, synovial hyperplasia. (b) Pathological scores of synovitis, pannus formation, degradation of cartilage, and bone were evaluated blindly (*n* = 8). ^#^*P* < 0.05 and ^##^*P* < 0.01 compared to the CIA group.

We observed severe inflammatory infiltration, extended cartilage destruction, and bone erosion in the CIA group (Figure 3(a)). In the EB group, the synovial tissue of the ankle joint showed pannus formation and synovial hyperplasia. The EF and TG groups showed a remarkable reduction in synovial hyperplasia, inflammatory cell infiltration, and pannus formation compared to that in CIA rats. The improvement of bone erosion in EB, EF, and TG group was significantly better than that in EL group. EL treatment reduced synovial hyperplasia and immune cell infiltration, but a few pannus layers were observed. The pathological scores of synovitis, pannus formation, and degradation of cartilage and bone of all groups showed a significant improvement compared to those of the CIA group (Figure 3(b), *P* < 0.05).

### 3.4. Effects of EB, EL, and EF on Bone Destruction

**Figure 4:**
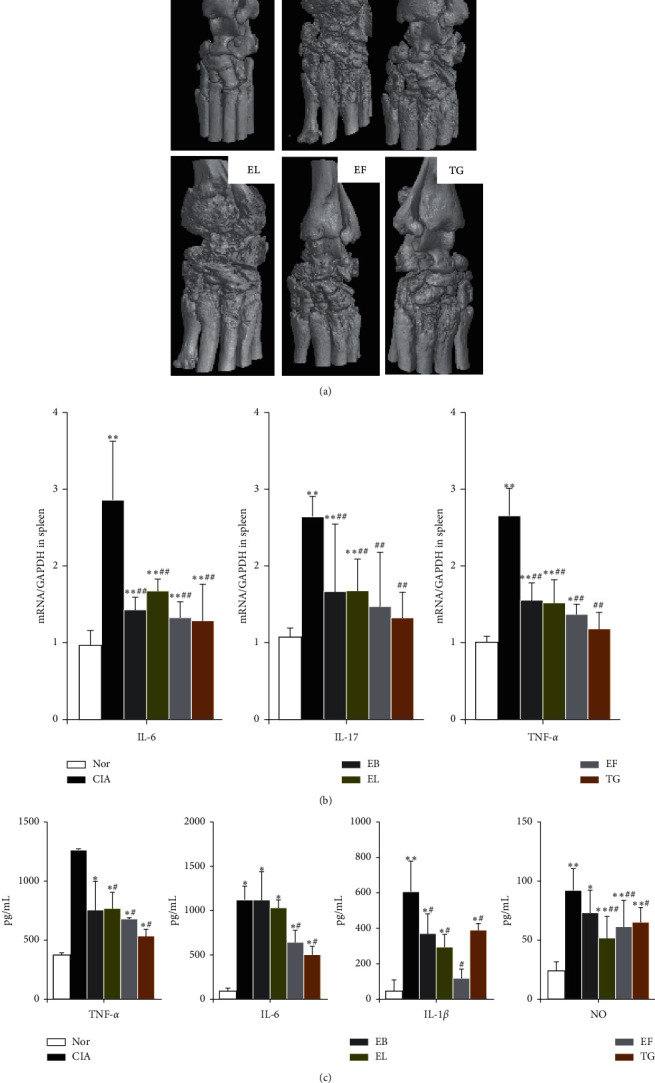
Efficacy of *Eucommia ulmoides* extracts against ankle joint destruction of collagen-induced arthritis (CIA) rats and inflammatory cytokine expression in the spleen and serum. (a) Representative microcomputed tomography (CT) images of ankle joints of all groups (*n* = 8). (b) mRNA expression levels of IL-6, IL-17, and TNF*-α* in rat spleens determined using quantitative reverse transcription-polymerase chain reactions (qRT-PCR, *n* = 8). ^*∗*^*P* < 0.05 and ^*∗∗*^*P* < 0.01 compared to Nor group, ^#^*P* < 0.05 and ^##^*P* < 0.01 compared to CIA group. (c) Serum levels of interleukin- (IL-) 1*β*, tumor necrosis factor- (TNF-) *α*, IL-6, and nitric oxide (NO, *n* = 8).

Micro-CT scanning clearly revealed the effect of the EB, EL, and EF on the ankle joint structure of rats. The ankle joint surface of the Nor group was smooth and free of bone damage (Figure 4(a)). In the CIA group, the ankle bone erosion was severe and had a honeycomb appearance. The degree of ankle joint destruction in rats in all treatment groups showed significant improvements compared to those in the CIA group.

### 3.5. Effects of EB, EL, and EF on Inflammatory Cytokines

Serum IL-1*β*, TNF-*α*, and NO levels were significantly lower in the EL group (*P* < 0.05) than in the other groups. *IL-6*, *IL-17*, and *TNF-α* mRNA levels in the spleen were significantly higher in the CIA group than in the Nor group (*P* < 0.01, Figure 4(b)). Importantly, treatment with EB, EL, EF, and TG significantly suppressed the expression of all three cytokines (*P* < 0.05). Serum IL-1*β*, TNF-*α*, IL-6, and NO concentrations were significantly higher in the CIA group than in the Nor group (*P* < 0.05). The expression of these cytokines was significantly reduced only in the EF and TG groups (*P* < 0.05). Serum IL-1*β* level was significantly decreased in the EB group compared to that in the other groups (Figure 4(c)).

## 4. Discussion

RA is characterized by chronic and progressive organ inflammation causing joint destruction, decreased life expectancy, and reduced quality of life [23, 24]. During joint destruction, many immune cells are localized to the synovial tissue and inflammatory cells are immersed in the lubricating membrane cavity, thereby activating synovial microvascular endothelial cells to promote cell migration and increase adhesion molecule and chemokine expression [25–27]. These changes in the microenvironment induce synovial structural reorganization and local fibroblast activation, resulting in RA synovial tissue formation, ultimately leading to joint swelling, stiffness, deformity, and dysfunction.

TNF-*α* can increase the number of osteoclast precursor cells and induce osteoclast differentiation of macrophages in the bone marrow and maturation of osteoclasts for the promotion of bone resorption [28]. Cytokines of the IL-1 family (including IL-1*α*, −1*β*, −18, and −33) are abundantly expressed in RA joints, ultimately promoting the activation of leukocytes, endothelial cells, chondrocytes, and osteoclasts. The role of IL-1*β* in the stimulation of inflammation and joint erosion has been determined using *in vitro* and *in vivo* experiments [29, 30]. Ethanol extracts of *E. ulmoides* leaves inhibited serum IL-6 and TNF-*α* expression in ovariectomized rats by regulating cytokine expression to reduce osteoclast proliferation, inhibit bone resorption, reduce bone destruction, regulate bone metabolism balance, and increase bone density [31, 32]. Similarly, the present study showed that the alcohol extracts of different parts of *E. ulmoides* inhibited the swelling of ankle joints of CIA rats. Histopathological analysis revealed the pharmacological effects of the EB, EL, and EL extracts, which significantly diminished synovial hyperplasia and improved bone erosion in RA rats. Studies have shown that *E. ulmoides* reduces IL-1*β* and TNF-*α* levels in CIA mouse serum [33].

IL-17 is a major inflammatory cytokine that plays a key role in tissue inflammation, autoimmunity, and host defense mechanisms. High IL-17 levels have been detected in the synovial fluid of RA patients [34]. *In vivo* experiments showed that IL-17 expression was suppressed by administering EL and EF. NO is involved in autoimmune-mediated tissue destruction and inflammation. During arthritis development, inflammatory cells produce NO, which is mediated by cytokine activity. A high NO concentration is extremely cytotoxic, causing tissue and joint damage with different degrees of lesioning. NO may also be directly involved in the pathogenesis of the RA inflammatory response by acting as an inflammatory mediator [35]. *In vitro* experiments showed that EB, EL, and EF significantly inhibited the expression of NO, which was also suppressed by EL and EF in *in vivo* experiments.

Differences in the types and content of the chemical constituents of *Eucommia* bark, leaves, and male flowers have been previously reported [36]. *Eucommia* bark mainly contains genipin, rosinol diglucoside, and aucubin while the leaves mainly contain chlorogenic acid, quercetin, aucubin, and genipin. The male flowers contain chlorogenic acid, geniposidic acid, and genipin [5, 37].

In this study, we also found that the extracts of all three components had different effects on inflammatory factors both *in vitro* and *in vivo*. We found that several factors affect the expression of inflammatory factors *in vivo* and that extracts from all three plant components of *E. ulmoides* had different effects on CIA. Administration of EB, EL, and EF gradually reduced the degree of plantar swelling, indicating that all extracts had inhibitory effects on RA. Histopathological examination of the tissue sections and determination of inflammatory factors revealed that the anti-inflammatory effect of EB was not as potent as that of EL and EF, but the inhibition of joint osteoclasts by EB was significantly better than that induced by EL and EF. Histopathological analysis of the tissue slices combined with the micro-CT scan results showed that there was less bone erosion in the EB group than there was in the EL and EF groups. Studies have shown that the rosin diglucoside in *E. ulmoides* can inhibit osteoporosis by promoting the proliferation and differentiation of osteoblasts [38]. Therefore, we concluded that EB exhibited the most potent bone-protective effect, which could be attributed to the presence of the pine resin, glucosinolate.

EL showed more superior anti-inflammatory effects than EB *in vivo*, likely because EL mainly contains genipin, chlorogenic acid, quercetin, and other active chemical components [37]. The pharmacological effect of *E. ulmoides* leaves and bark is reported to be similar, providing an experimental basis and evidence to support the medicinal use of *E. ulmoides* leaves instead of the bark [39]. Collectively, the pharmacological benefits of EF may likely be attributed to its constituents, chlorogenic acid, geniposide, and aucubin. Chlorogenic acid present in *E. ulmoides* has an anti-inflammatory effect. This effect combined with the analgesic and bacteriostatic effects of the male flowers of *E. ulmoides* is superior to those of the bark and leaves [13, 40].

## 5. Conclusions

The extracts of the bark, leaves, and male flowers of *E. ulmoides* improved the plantar swelling and arthritis index of CIA rats, inhibited synovial hyperplasia and pannus formation, and reduced bone erosion. These findings showed that the three components of *E. ulmoides* alleviated inflammation and bone destruction in the CIA rat model. However, extracts of the three plant parts showed differing degrees of anti-inflammatory effects. EB exhibited the most potent inhibition of bone erosion, EL displayed better anti-inflammatory effects than EB, and EF exhibited the highest inhibition of inflammatory tissue infiltration and pannus formation of joint tissue. Therefore, further research should be conducted on the mechanism of action of these extracts.

## Data Availability

The research data used to support the findings of this study are available from the corresponding author upon request.
